# Integrase Inhibitor Resistance Mechanisms and Structural Characteristics in Antiretroviral Therapy-Experienced, Integrase Inhibitor-Naive Adults with HIV-1 Infection Treated with Dolutegravir plus Two Nucleoside Reverse Transcriptase Inhibitors in the DAWNING Study

**DOI:** 10.1128/AAC.01643-21

**Published:** 2022-01-18

**Authors:** Mark Underwood, Joe Horton, Keith Nangle, Judy Hopking, Kimberly Smith, Michael Aboud, Brian Wynne, Jörg Sievers, Eugene L. Stewart, Ruolan Wang

**Affiliations:** a ViiV Healthcare, Research Triangle Park, North Carolina, USA; b Parexel International, Durham, North Carolina, USA; c GlaxoSmithKline, Stockley Park, United Kingdom; d ViiV Healthcare, Brentford, United Kingdom; e GlaxoSmithKline, Upper Providence, Pennsylvania, USA

**Keywords:** HIV-1 infection, integrase strand transfer inhibitor, antiretroviral agents, barrier to resistance, dolutegravir

## Abstract

At week 48 in the phase IIIb DAWNING study, the integrase strand transfer inhibitor (INSTI) dolutegravir plus 2 nucleoside reverse transcriptase inhibitors demonstrated superiority to ritonavir-boosted lopinavir in achieving virologic suppression in adults with HIV-1 who failed first-line therapy. Here, we report emergent HIV-1 drug resistance and mechanistic underpinnings among dolutegravir-treated adults in DAWNING. Population viral genotyping, phenotyping, and clonal analyses were performed on participants meeting confirmed virologic withdrawal (CVW) criteria on dolutegravir-containing regimens. Dolutegravir binding to and structural changes in HIV-1 integrase-DNA complexes with INSTI resistance-associated substitutions were evaluated. Of participants who received dolutegravir through week 48 plus an additional 110 weeks for this assessment, 6 met CVW criteria with treatment-emergent INSTI resistance-associated substitutions and 1 had R263R/K at baseline but not at CVW. All 7 achieved HIV-1 RNA levels of <400 copies/mL (5 achieved <50 copies/mL) before CVW. Treatment-emergent G118R was detected in 5 participants, occurring with ≥2 other integrase substitutions, including R263R/K, in 3 participants and without other integrase substitutions in 2 participants. G118R or R263K increased the rate of dolutegravir dissociation from integrase-DNA complexes versus wild-type but retained prolonged binding. Overall, among treatment-experienced adults who received dolutegravir in DAWNING, 6 of 314 participants developed treatment-emergent INSTI resistance-associated substitutions, with a change in *in vitro* dolutegravir resistance of >10-fold and reduced viral replication capacity versus baseline levels. This study demonstrates that the pathway to dolutegravir resistance is a challenging balance between HIV-1 phenotypic change and associated loss of viral fitness. (This study has been registered at ClinicalTrials.gov under identifier NCT02227238.)

## INTRODUCTION

Dolutegravir is a second-generation integrase strand transfer inhibitor (INSTI) with a high barrier to resistance and an associated resistance profile distinct from those of the first-generation INSTIs raltegravir and elvitegravir (1–4). In several phase III studies, dolutegravir demonstrated virologic efficacy noninferior or superior to those of drugs in the INSTI (1, 5), nonnucleoside reverse transcriptase inhibitor (NNRTI) (2), and protease inhibitor (PI) (6, 7) classes. The proportions of participants with treatment failure have been low (1, 2, 5–8), and only participants with preexisting virologic failure with resistance to antiretroviral drugs failed with treatment-emergent dolutegravir resistance while on 3-drug dolutegravir-containing regimens (1, 9).

The phase IIIb DAWNING study evaluated the antiviral efficacy and safety of dolutegravir compared with ritonavir-boosted lopinavir, both in combination with 2 nucleoside reverse transcriptase inhibitors (NRTIs), in adults with HIV-1 infection who had virologic failure on a first-line antiretroviral therapy (ART) regimen (9). At week 48, dolutegravir demonstrated superiority to ritonavir-boosted lopinavir in achieving virologic suppression (HIV-1 RNA levels of <50 copies/mL), and fewer participants met criteria for confirmed virologic withdrawal (CVW) with dolutegravir (11 [4%] of 314) versus ritonavir-boosted lopinavir (30 [10%] of 310). In fulfillment of the independent data monitoring committee recommendation after an *ad hoc* review was conducted, including data from 98% of participants through week 24, 12 participants receiving ritonavir-boosted lopinavir switched to dolutegravir in combination with 2 NRTIs and continued to participate during the continuation phase. Findings from the DAWNING study supported the World Health Organization’s recommendation of dolutegravir in combination with an optimized NRTI backbone over boosted PIs as the preferred second-line ART regimen in people with HIV failing a first-line NNRTI-based regimen (10).

Here, we present a *post hoc* analysis of participants who met CVW criteria and had INSTI resistance in the DAWNING study. Mechanisms and characteristics of INSTI resistance, including clonal analysis of integrase R263 and G118 pathway isolates, dolutegravir dissociation from integrase-DNA complexes, and integrase structural analysis, are described.

## RESULTS

### Participants meeting CVW criteria.

Through the week 48 analysis, 11 of 314 participants who received dolutegravir and 30 of 310 participants who received ritonavir-boosted lopinavir met CVW criteria, with 2 of 11 participants who received dolutegravir developing treatment-emergent genotypic and phenotypic INSTI resistance (9).

**FIG 1:**
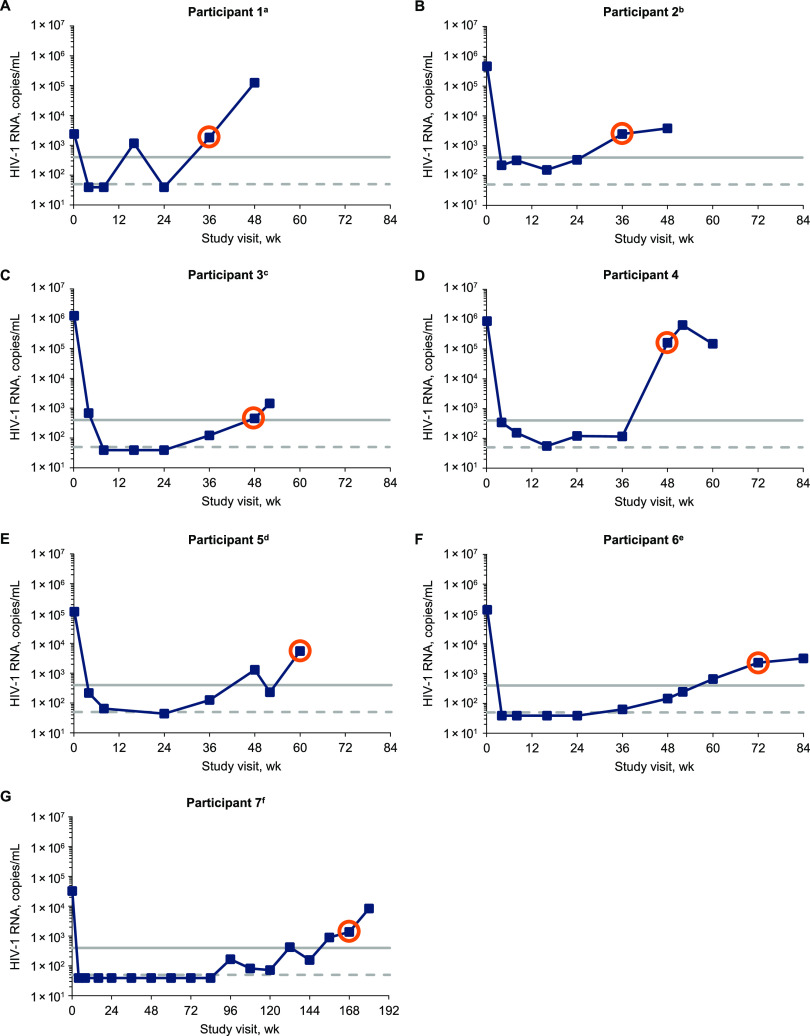
HIV-1 RNA over time in participants with CVW and INSTI resistance-associated substitutions. Orange circles denote viral load at the week each participant met CVW criteria and also indicate the on-study time point for samples used for population resistance testing and clonal analyses for participants 1 through 3. HIV-1 RNA levels of 400 and 50 copies/mL are indicated by solid and dashed gray lines, respectively. Study visits with HIV-1 RNA <40 copies/mL were plotted as 39 copies/mL. a, the repeat HIV-1 RNA levels were <40 copies/mL at week 24 and 19,486 copies/mL at week 48. b, the repeat HIV-1 RNA level was 3,526 copies/mL at week 36. c, the repeat HIV-1 RNA level was 1,589 copies/mL at week 52. d, the repeat HIV-1 RNA levels were 101 copies/mL at week 52 and 3,011 and 4,146 copies/mL at week 60. e, the repeat HIV-1 RNA levels were 322 copies/mL at week 60 and 6,692 copies/mL at week 72. f, the repeat HIV-1 RNA levels were 203 copies/mL at week 144, 274 copies/mL at week 156, 1,485 copies/mL at week 168, and 12,028 copies/mL at week 180.

This resistance analysis assessed all participants who met CVW criteria and had INSTI resistance while receiving dolutegravir through 158 weeks. In addition to the 2 participants who developed INSTI resistance with dolutegravir through week 48, 5 participants met CVW criteria and developed INSTI resistance after week 48, resulting in a total of 7 participants included in this analysis. Of participants meeting CVW criteria, all reached HIV-1 RNA levels of <400 copies/mL and 5 achieved levels of <50 copies/mL (Fig. 1). Of the 7 participants with INSTI resistance in the dolutegravir group from day 1, participant 1 had resistance-associated integrase substitutions at baseline and participants 2 through 7 had resistance-associated integrase substitutions at CVW (Table 1). None of the 12 participants who switched from ritonavir-boosted lopinavir to the dolutegravir regimen at week 48 had INSTI resistance present. Five of the 7 participants had previously received efavirenz plus tenofovir disoproxil fumarate with either emtricitabine (3 participants) or lamivudine (2 participants) at screening and through randomization, and the other 2 participants had received nevirapine plus lamivudine with either zidovudine or tenofovir disoproxil fumarate (Table 2). The background ART regimen for each participant at time of CVW with a dolutegravir-based regimen was either lamivudine plus zidovudine (6 participants) or emtricitabine plus tenofovir disoproxil fumarate (1 participant).

**TABLE 1:** Summary of virologic and resistance characteristics at baseline and CVW in participants with INSTI resistance-associated substitutions^*a*^

Patient	Study visit	HIV-1 subtype^*b*^	HIV-1 RNA level (copies/mL)	Replication capacity (%)^*c*^	Fold change in dolutegravir sensitivity^*d*^	INSTI resistance mutation(s)^*e*^^,^^*f*^	NRTI resistance mutation(s)^*e*^^,^^*g*^
1	Baseline	C	2,395	41	1.03	R263R/K	K65R, M184I/V
	Wk 36		1,823	69	0.79		
2	Baseline	B	461,801	103	1.02		M184V, K219K/E
	Wk 36		2,464	5.6	30	**G118R**	**D67N**, M184V
3	Baseline	C	1,248,517	236	1.26		K70E, M184V
	Wk 48		454	36	15	**H51H/Y**, **G118R**, **E138E/K**, **263R/K**	M184V
4	Baseline	A1	852,142	151	0.93	L74I	K65R, Y115F, M184V
	Wk 48		159,223	27	20	L74I, **G118G/R**, **E138E/K**, **148Q/R**, **R263R/K**	
5	Baseline	Complex	114,903	53	0.59		K65R, M184V
	Wk 60		5,479	27	>106.45	**E138K**, **G140S**, **Q148H**, **N155H**	M184V
6	Baseline	C	137,838	117	0.6	L74L/I	A62A/V, K65R, M184V
	Wk 72		2,332	20	22	**T66T/I**, **L74I**, **G118R**, **E138E/K**	A62A/V, M184V
7	Baseline	C	32,376	ND	0.98		K70E, Y115F, M184V
	Wk 168		1,380	ND	28	**G118R**	M184V

a INSTI, integrase strand transfer inhibitor; ND, not determinable by assay; NRTI, nucleoside reverse transcriptase inhibitor.

b Determined from reverse transcriptase and protease regions by Monogram Biosciences genotype assay.

c Determined from integrase region by Monogram Biosciences using the PhenoSense Integrase assay. Values are relative to that for the wild-type laboratory strain.

d Fold change in half-maximal inhibitory concentration relative to the wild-type value. The clinical cutoff is 4.0.

e Treatment-emergent substitutions are in bold.

f From the following prespecified integrase substitution list: H51Y, T66A/I/K, L68V/I, L74M/I, E92Q/V/G, Q95K, T97A, G118R, F121Y, E138A/K/D/T, G140A/C/S, Y143C/H/R/K/S/G/A, P145S, Q146P, S147G, Q148H/K/R, V151I/L/A, S153F/Y, N155H/S/T, E157Q, G163R/K, S230R, R263K, and G193E.

g International Antiviral Society (USA) major substitutions (34).

**TABLE 2:** Prior ART duration and background ART regimen in participants with INSTI resistance-associated substitutions^*a*^

Patient	Prior ART discontinued before screening		ART taken at screening until randomization		Background ART regimen on study^*b*^
	ART agent(s)	Duration (wks)	ART agent(s)	Duration (wks)	
1	3TC, NVP, TDF	30	EFV + FTC + TDF	81	3TC + ZDV
2			3TC + ZDV, NVP	55	FTC + TDF
3			EFV + FTC + TDF	49	3TC + ZDV
4			EFV, 3TC, TDF	41	3TC + ZDV
5			EFV + 3TC + TDF	211	3TC + ZDV
6			EFV + FTC + TDF	31	3TC + ZDV
7	EFV	115	3TC	554	3TC + ZDV
	D4T	403	NVP	491	
			TDF	152	

a ART, antiretroviral therapy; D4T, stavudine; EFV, efavirenz; FTC, emtricitabine; NVP, nevirapine; 3TC, lamivudine; TDF, tenofovir disoproxil fumarate; ZDV, zidovudine.

b 3TC and FTC were inactive agents in these participants.

### Genotypic analysis.

Of the 7 participants with INSTI resistance-associated substitutions, 4 had HIV-1 subtype C, all of whom were from South Africa; the other 3 participants had B, A1, and complex subtypes and were from Colombia, Ukraine, and Brazil, respectively (Table 1). During the study, each participant received a background regimen containing either lamivudine or emtricitabine, which were inactive in these participants. One participant (participant 1) had the integrase substitution R263R/K and NRTI resistance-associated substitutions K65R and M184I/V at baseline, despite no apparent prior INSTI treatment, but did not demonstrate *in vitro* dolutegravir phenotypic resistance. At CVW, this participant lost the R263R/K integrase substitution, had no other INSTI or NRTI resistance-associated substitutions, and did not demonstrate genotypic or phenotypic dolutegravir resistance. The remaining 6 participants had treatment-emergent INSTI resistance-associated substitutions, 1 of whom was taking tenofovir disoproxil fumarate and 5 of whom were taking zidovudine as the active background ART agent. Five participants had G118R, which emerged in combination with other integrase substitutions in 3 participants or alone in 2 participants. Two of 3 participants with G118R plus other integrase substitutions also had treatment-emergent R263R/K. The sixth participant had treatment-emergent Q148H and N155H in combination with other integrase substitutions. Each participant with treatment-emergent integrase substitutions demonstrated *in vitro* resistance to dolutegravir at CVW (median [range] fold change in dolutegravir susceptibility at CVW, 25 [15 to >106.45]). The NRTI resistance-associated substitution M184V was detected at baseline in all 7 participants, 5 of whom retained this substitution at CVW and 2 of whom had no NRTI resistance-associated substitutions at CVW. The treatment-emergent NRTI resistance-associated substitution D67N was observed in 1 participant. Of 6 participants with treatment-emergent INSTI resistance-associated substitutions, 5 had available replication capacity results at CVW, all of whom demonstrated decreased replication capacity at CVW versus baseline.

### Clonal phylogenetic, genotypic, and phenotypic analysis.

**FIG 2:**
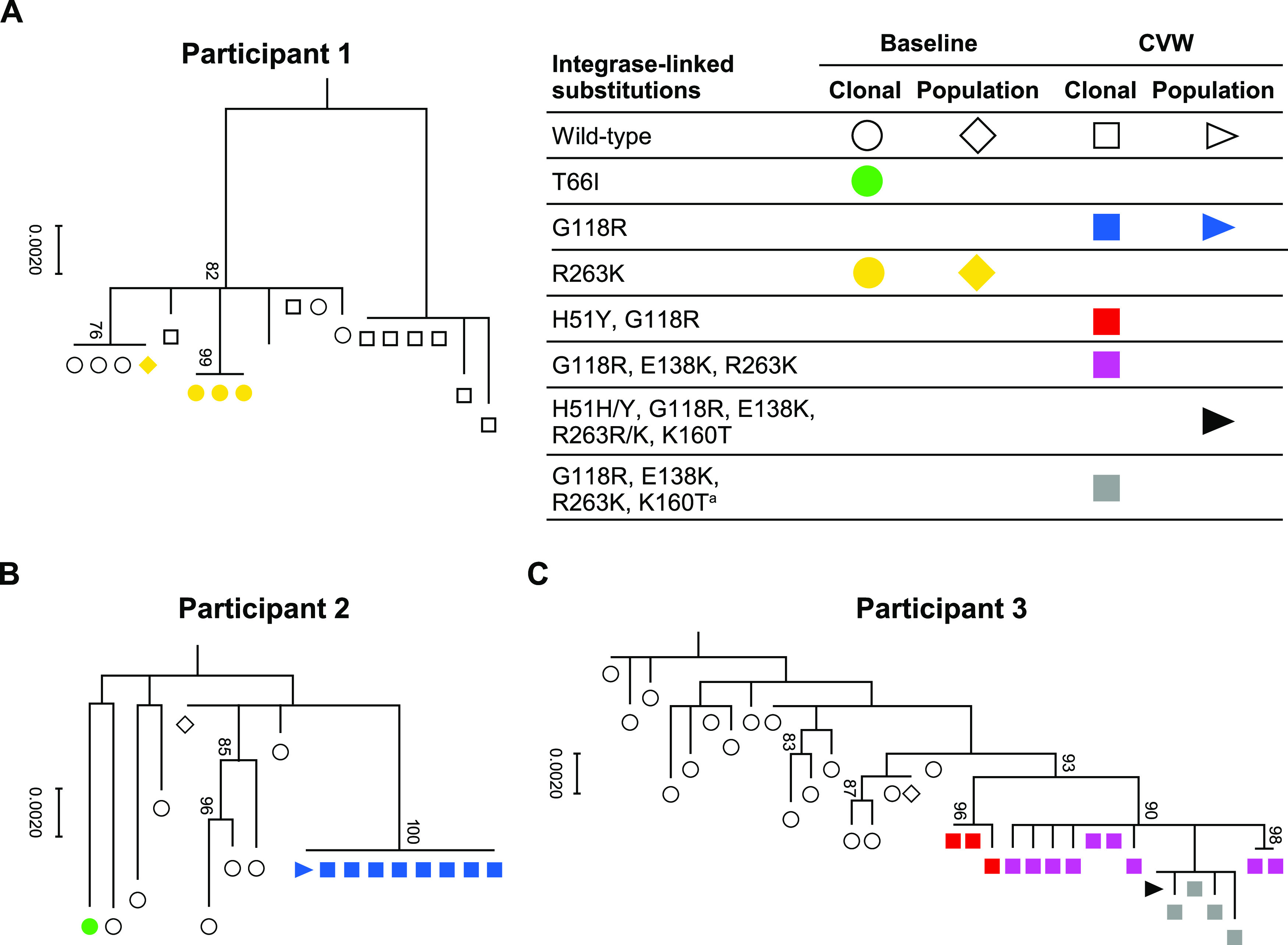
Phylogenetic analysis of variant clonal integrase sequences at baseline and CVW. Samples used for clonal analyses at CVW time points are presented in Fig. 1 and are the same samples used for the corresponding population-level resistance data reported in Table 1. Bootstrap confidence levels are indicated on each diagram. CVW, confirmed virologic withdrawal. a, K160T was observed in 4 of 13 clones containing G118R, E138K, and R263K integrase substitutions but is not a prespecified dolutegravir resistance-associated substitution (34).

Results from the phylogenetic analysis showed a common ancestry for each of the clonal and population clusters from each participant (Fig. 2). Clonal and population sequences from participant 1 formed 2 main clusters (Fig. 2A). One cluster contained all baseline sequences with R263 (bootstrap = 82%), and the other cluster contained only wild-type CVW sequences; all baseline clones containing R263K were identical at the nucleotide level. In the phylogenetic trees for CVW participants 2 and 3, distinct clusters containing only post-baseline sequences formed, each with high bootstrap values, 100% and 93%, respectively. All CVW clonal and population sequences from participant 2 clustered together; all sequences contained G118R and were identical at the nucleotide level (Fig. 2B). Clonal and population sequences at CVW from participant 3 had multiple evolving pathways with ≥2 integrase substitutions, with separate clusters forming for sequences containing H51Y and G118R (bootstrap = 96%) and those containing G118R, E138K, and R263K (bootstrap = 90%) (Fig. 2C). A subcluster of sequences containing K160T in addition to G118R, E138K, and R263K showed the greatest evolutionary distance.

**FIG 3:**
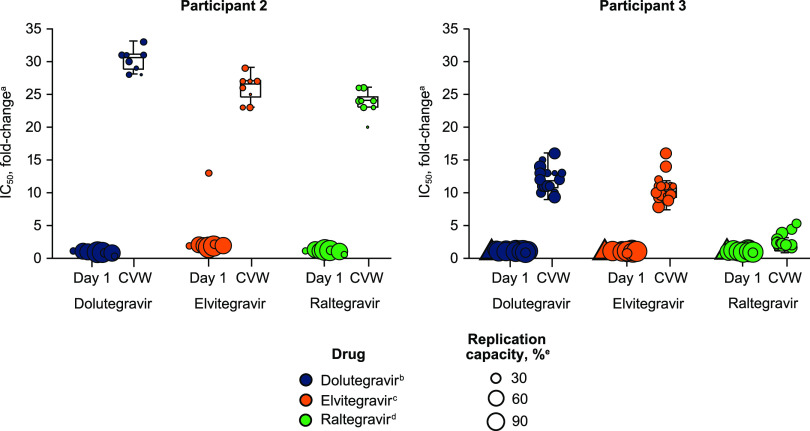
Variant clone drug sensitivity and integrase region-based replication capacity at baseline and CVW. Replication capacity values for each variant clone are represented by symbol size. Variant clones missing data for replication capacity are indicated by triangles. CVW, confirmed virologic withdrawal; IC_50_, half-maximal inhibitory concentration. a, values are relative to the wild type. b, the clinical cutoff for dolutegravir is 4.0. c, the biological cutoff for elvitegravir is 2.5. d, the biological cutoff for raltegravir is 1.5. e, values are relative to wild-type replication.

Wild-type variant clones from participant 1 demonstrated similar INSTI sensitivity and replication capacity at baseline and CVW (Table 3). Baseline clones from participant 1 with R263K had reduced replication capacity compared with wild-type clones but demonstrated *in vitro* resistance only to elvitegravir, not dolutegravir or raltegravir. Variant clones from participants 2 and 3 demonstrated increased resistance to dolutegravir, elvitegravir, and raltegravir and reduced replication capacity at CVW compared with baseline clones (Fig. 3; Table 3). Clones from participant 2 with G118R alone had increased INSTI resistance and decreased replication capacity compared with clones from participant 3 with G118R plus other integrase substitutions (Table 3). Median drug sensitivity and replication capacity were similar for clones from participant 3 with G118R plus H51Y versus clones with G118R plus E138K and R263K.

**TABLE 3:** Variant clone drug sensitivity and replication capacity at baseline and CVW

Patient	Study visit^*a*^	Integrase-linked substitution(s)	No. of clones	Fold change in median drug sensitivity^*b*^			Median replication capacity (%)^*c*^
				Dolutegravir^*d*^	Elvitegravir^*e*^	Raltegravir^*f*^	
1	Baseline	Wild-type	5	0.87	1	0.88	81
		R263K	3	1.81	2.85	1.09	27
	CVW (wk 36)	Wild-type	8	0.77	0.86	0.89	78
2	Baseline	Wild-type	7	0.93	1.94	1.18	67
		T66I	1	0.33	13	0.58	12
	CVW (wk 36)	G118R	8	30.5	26.5	24	9.55
3	Baseline	Wild-type	16	1.08	1.03	1.03	91
	CVW (wk 48)	H51Y, G118R	3	11	10	4.43	28
		G118R, E138K, R263K	13	13	11	2.03	27

a CVW, confirmed virologic withdrawal.

b Fold change in half-maximal inhibitory concentration relative to the wild-type value.

c Values are relative to wild-type replication.

d The clinical cutoff for dolutegravir is 4.0.

e The biological cutoff for elvitegravir is 2.5.

f The biological cutoff for raltegravir is 1.5.

### Effect of integrase substitutions on INSTI dissociation and integrase structure.

Dolutegravir dissociated from R263K integrase-DNA complexes faster than from wild-type integrase-DNA complexes, with a 1.8-fold increase in dissociation rate constant (*k*_off_) (Table 4). Integrase mutants with G118R or G118R plus E138K exhibited faster dolutegravir dissociation than those with R263K, with 8.6- and 9.0-fold increases in *k*_off_ values, respectively, relative to the wild type. Half of the dolutegravir bound to integrase-DNA complexes containing either R263K or G118R substitutions was retained at ∼50 and ∼10 h, respectively. The dissociation of raltegravir and elvitegravir from the G118R integrase-DNA complex was faster than that of the wild type, with 3.4- and 2.9-fold increases in *k*_off_, respectively, and demonstrated substantially shorter binding, with dissociative half-lives of 2.7 and 0.9 h, respectively. The R263K substitution appeared to have little effect on dissociation of raltegravir (no change in *k*_off_) and elvitegravir (1.2-fold increase in *k*_off_) from integrase-DNA complexes relative to the wild type.

**TABLE 4:** Dissociation of INSTIs from integrase-DNA complexes with INSTI resistance-associated substitutions^*a*^

Integrase-linked substitution(s)	*k*_off_ (10^−6^ s^−1^) (mean ± SD)			*t*_1/2_ (h)^*b*^		
	Dolutegravir	Elvitegravir	Raltegravir	Dolutegravir	Elvitegravir	Raltegravir
Wild-type^*c*^	2.1 ± 0.1	75 ± 9	21 ± 2	92	2.6	9.2
R263K^*c*^	3.7 ± 0.2	89 ± 11	20 ± 3	52	2.2	9.6
						
G118R^*d*^	18 ± 1	215 ± 11	71 ± 12	10.7	0.9	2.7
G118R, E138K^*d*^	19 ± 1	168 ± 6	61 ± 3	10.1	1.1	3.2

a *k*_off_, dissociation rate constant; *t*_1/2_, half-life.

b For reference, *t*_1/2_ values for dolutegravir, elvitegravir, and raltegravir with a wild-type integrase-DNA complex were previously measured to be 71, 2.7, and 8.8 h, respectively (35).

c *k*_off_ values represent data from 3 or 4 independent experiments.

d *k*_off_ values represent data from 3 to 7 independent experiments.

**FIG 4:**
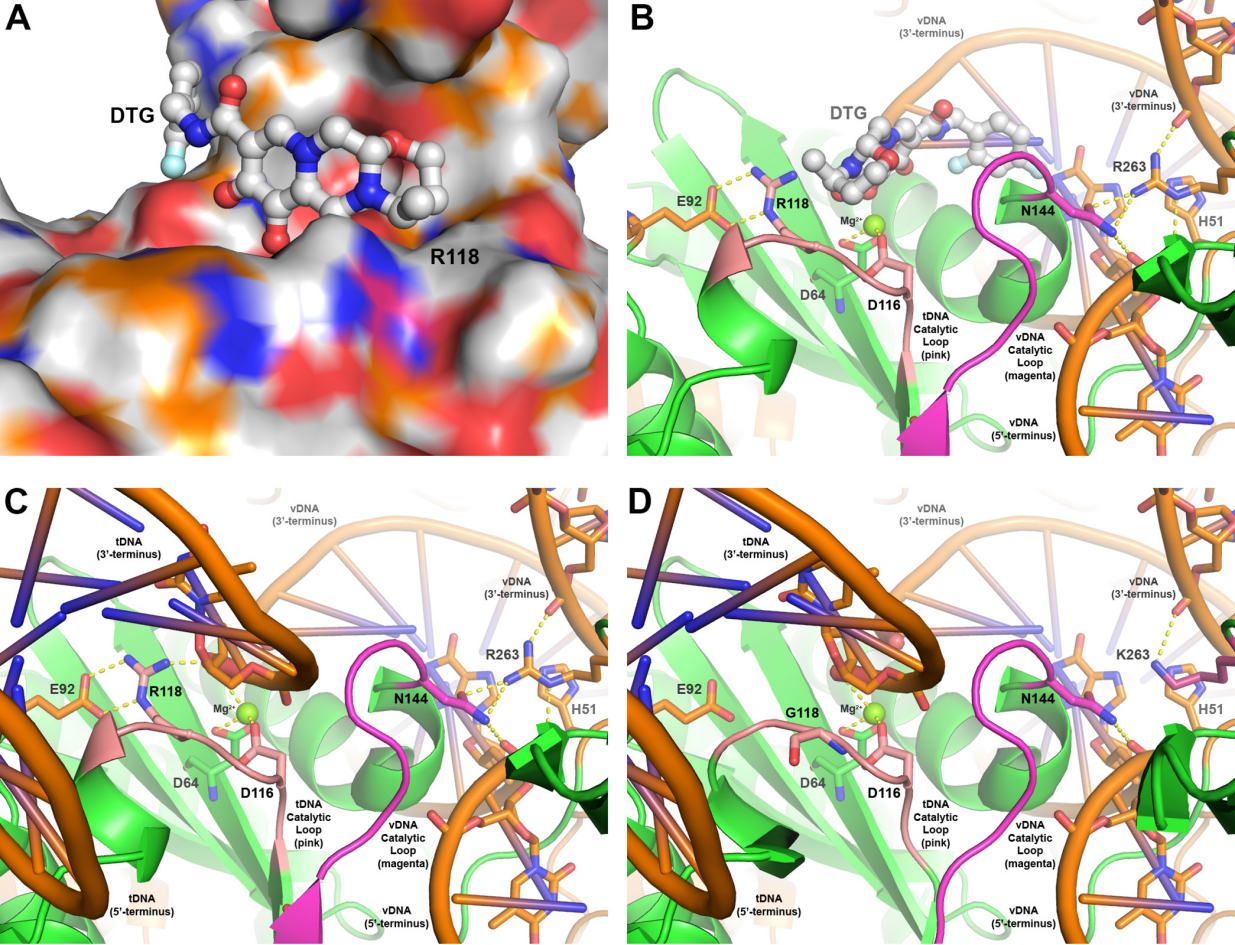
Models of HIV-1 integrase containing wild-type and/or mutant amino acids in the intasome complex. (A) Surface view of the integrase G118R mutant catalytic site (colored by atom with carbons in orange) bound with dolutegravir (rendered in ball-and-stick format and colored by atom with carbons in white). (B) Ribbon-and-stick rendering of the integrase G118R mutant catalytic site (in green) bound with vDNA (in orange). Dolutegravir (as rendered in panel A) binds between the vDNA and tDNA catalytic loops and interacts with the catalytic Mg^2+^ (rendered in ball-and-stick format and colored in green). The tDNA and vDNA catalytic loops are highlighted in pink and magenta, respectively, and illustrate the locations of G118 and N144 (rendered in stick format) on each loop. Hydrogen bonding interactions between N144 with R263 and G118R with E92 are indicated by dashed yellow lines. (C) The integrase G118R mutant catalytic site (as rendered and oriented in panel B) bound to both vDNA and tDNA substrates (in orange). Hydrogen bonding interactions among G118R, the 3′ terminus of the tDNA, and the Mg^2+^ are indicated by dashed yellow lines. (D) Ribbon-and-stick rendering of the integrase catalytic site containing R263K (colored in magenta) and wild-type G118 bound with vDNA and tDNA substrates. DTG, dolutegravir; tDNA, host target DNA; vDNA, viral DNA.

In an effort to understand the emerging HIV-1 resistance mutants and their associated kinetics at a molecular level, we developed 2 HIV-1 integrase homology models containing either G118R or R263K based on the cryogenic electron microscopy (cryo-EM) intasome structure reported in the literature (11). The homology model of the HIV-1 integrase G118R resistance mutant revealed that G118R partially occludes the integrase catalytic binding site, which would prevent dolutegravir and other INSTIs from binding effectively and cause dissociation from the HIV-1 intasome to be more rapid (Fig. 4A; see Text S1 and Movie S1 in the supplemental material for additional details). When bound with both viral DNA (vDNA) and host target DNA (tDNA), G118R interacts with the 5′ phosphate of the tDNA catalytic adenosine (Fig. 4B and C). In addition, G118R forms a dual hydrogen bond with E92 and a hydrogen bond with the 3′ hydroxy of the tDNA terminal thymine when bound with both vDNA and tDNA. These additional hydrogen bonding interactions are unobserved in HIV-1 integrase containing the wild-type amino acid G118 (Fig. 4D). Structural analysis of HIV-1 integrase containing the wild-type amino acid R263 bound with vDNA shows that R263 forms multiple hydrogen bonds among the catalytic loop, including a dual hydrogen bond with N144 and with both the 3′ and 5′ strands of the vDNA (Fig. 4B and C). In the integrase R263K mutant homology model, all but one hydrogen bond with the substrate and catalytic loop were eliminated (Fig. 4D), resulting in differential geometry of the catalytic site relative to HIV-1 integrase containing the wild-type R263 amino acid.

## DISCUSSION

Emergence of INSTI resistance-associated substitutions among participants in the DAWNING study was observed in 6 (2%) of 314 participants receiving dolutegravir plus 2 NRTIs from day 1, with the latest occurrence at week 168, and in none of the 12 participants who switched to the dolutegravir-based regimen from ritonavir-boosted lopinavir after week 48. Six participants received zidovudine as the active background agent, and all had M184V resistance substitutions with prior lamivudine or emtricitabine treatment. The results are consistent with results from the SAILING study, in which treatment-emergent INSTI resistance occurred in 7 (2%) of 354 ART-experienced participants with multiclass resistance who received dolutegravir in combination with 1 or 2 active background agents through and after week 48 (1, 12). Both the DAWNING and SAILING studies had a 48-week primary endpoint followed by a continuation phase for several years during which resistance continued to be monitored (1, 9). One participant in DAWNING had emergence of the integrase substitutions E138K, G140S, Q148H, and N155H. Dolutegravir has selected for N155H, both alone and in combination with E138K and/or other integrase substitutions, in ART-experienced participants from several studies, including SAILING and multiple trials evaluating dolutegravir monotherapy (12–18). Treatment with raltegravir and elvitegravir frequently selects for G140S and Q148H in combination, although emergence of major INSTI resistance-associated substitutions together, such as Q148H and N155H, occurs infrequently in patients (19–21). It was demonstrated that mutations at integrase positions 148 and 155 did not coexist on the same viral genome during raltegravir phase III trials, likely because sufficiently high-level raltegravir resistance was achieved with the separate substitutions and because N155H plus Q148 conferred substantial reductions in HIV-1 replication capacity (19, 22, 23). Similar to the participant from the present study, G140S and Q148H also emerged together with E138K and N155H in a participant who received dolutegravir monotherapy (16).

The remaining 5 participants in the DAWNING study had treatment-emergent G118R. G118R emerged in combination with ≥3 other integrase substitutions in 3 participants, including with R263R/K plus E138E/K in 2 participants. Dolutegravir also selects for G118R or R263K *in vitro*, both alone and in combination with E138K (24–27). G118R has also emerged without other integrase substitutions in ART-experienced individuals receiving dolutegravir monotherapy (16). Treatment-emergent R263K has occurred alone in individuals receiving dolutegravir monotherapy or in combination with ART agents (1, 17, 18, 28, 29). In the SAILING study, R263K emerged alone in 2 participants and in combination with the integrase substitutions A49G plus S230R in one participant (1, 12). Emergence of G118R and R263K has been documented in 1 case report of an individual receiving dolutegravir plus 2 NRTIs who was treated for tuberculosis and diagnosed with immune reconstitution inflammatory syndrome (30), but these DAWNING clonal analyses provide the first known demonstration that G118R can exist with R263K on the same HIV-1 genome.

All participants with treatment-emergent integrase substitutions demonstrated increased dolutegravir resistance and an associated decrease in replication capacity at CVW compared with baseline. The clonal genotypic and phenotypic results here suggested that the addition of integrase substitutions, such as H51Y or E138K with or without R263K, may decrease the impact of G118R on INSTI resistance and provide increased HIV-1 replication capacity. It is further consistent with *in vitro* observations (31) that G118R substantially decreases integrase strand transfer efficiency, which is partially restored by the addition of either H51Y or E138K; these changes enhanced DNA binding and resulted in increased available integrase-DNA complexes. Similar observations of integrase substitutions accumulating were noted in pediatric patients with preexisting non-INSTI resistance who failed dolutegravir-containing regimens while experiencing adherence difficulties (32).

Unusually, 1 participant had the highly conserved integrase substitutions R263R/K and M184I/V as mixtures at baseline but not at CVW (33). The reason the baseline INSTI and NRTI resistance was missing at the CVW viral load elevation is unclear but may be consistent with nonadherence.

The phylogenetic analysis revealed that all variant clones from each participant evolved from a single common ancestor. Greater diversity was observed in sequences collected at baseline than in those at CVW, consistent with continued drug pressure and apparently nonevolving HIV-1. Variant clones from participant 2 evolved along a single pathway to contain only G118R substitutions, with all sequences being identical at CVW. In contrast, participant 3 exhibited multiple evolving substitution pathways that yielded 2 different clonal clusters, with none of the clones harboring only a single resistance substitution. These cases may reflect stochastic and highly individual situations (e.g., selective drug levels, access to additional substitutions by single- versus multiple-step codon changes) that can assist or hinder development of added integrase substitutions. One subcluster from participant 3 showed greater evolutionary distance with K160T added to G118R, E138K, and R263K. K160T has been detected in individuals who have received elvitegravir or raltegravir treatment, but it is not associated with INSTI resistance (https://hivdb.stanford.edu/cgi-bin/Mutations.cgi?Gene=IN; 34). Therefore, it is unlikely that the increased evolutionary distance observed for variant clones with K160T corresponded with increased dolutegravir resistance or decreased replication capacity compared with those without K160T.

We have shown previously that dolutegravir dissociated more slowly from wild-type or mutant integrase-DNA complexes than raltegravir or elvitegravir (35). Of note here, dolutegravir dissociation from integrase-DNA complexes occurred faster with R263K or G118R with or without E138K compared with wild-type integrase but binding remained prolonged, with a half-life of ∼50 and ∼10 h, respectively, compared with dissociative half-lives from wild-type integrase-DNA complexes of 8.8 and 2.7 h for raltegravir and elvitegravir, respectively (35). Even in the presence of integrase substitutions, dolutegravir remains bound to integrase-DNA complexes for a prolonged time compared with other INSTIs. These results are consistent with dolutegravir retention of HIV-1 inhibitory capacity in the presence of R263K or G118R integrase substitutions.

HIV-1 integrase homology models of the G118R mutant protein have been previously developed in the presence of vDNA, tDNA, and various inhibitors in an attempt to rationalize resistance profiles at a molecular level (31, 36). Similar to our findings, the previous G118R resistance model illustrated a dual hydrogen bonding interaction between G118R, located on the tDNA catalytic loop, and E92 (31). However, because that G118R model was based on the crystal structure of the prototype foamy virus integrase bound only to vDNA (37), the hydrogen bond between G118R and the tDNA substrate was not observed in the resulting model (31). Later, a collection of HIV-1 integrase homology models described many complex interactions involving the G118R mutant, including with Mg^2+^ bound in the catalytic site and with various catalytic site residues, depending on the bound or unbound state of inhibitors or substrates (36). Our G118R resistance model is similar to the initial model but reveals an additional hydrogen bonding interaction between G118R and the 3′-terminal thymidine of the tDNA substrate (Text S1 and Movie S1).

Our homology model provides evidence of 3 dominant mechanisms that may collectively result in G118R-based resistance. G118R can prevent dolutegravir and other INSTIs from binding effectively by sterically occupying and partially occluding the integrase catalytic binding site, which is supported by our kinetic binding studies. In addition, G118R may promote strand transfer and integration of the vDNA through its interactions with the vDNA 5′ phosphate of the catalytic adenosine. Finally, G118R stabilizes the product tDNA by forming a direct hydrogen bond with the 3′ hydroxy of the tDNA terminal thymine after final formation of the vDNA/tDNA substrate complex. Through each of these mechanisms, the G118R resistance mutant promotes and potentially stabilizes the vDNA/tDNA integration and strand transfer complex during the HIV-1 integrase catalytic process. Collectively, these 3 possible resistance mechanisms would result in a mechanistic bottleneck because the steric occupation of G118R in the catalytic site both prevents INSTI binding and hinders tDNA binding. Such a mechanistic bottleneck may explain the reduced replication capacity observed with the G118R resistance substitution.

Before resolution of the HIV-1 intasome complex by cryo-EM, modeling studies suggested that R263 may be involved in long-distance spatial orientation of the vDNA substrate during integration (24, 38). This study’s analyses revealed that R263 plays 2 key roles in the integration process. First, R263 forms direct hydrogen bonds with both the 3′ and 5′ termini of the vDNA, resulting in distal orientation of the vDNA for integration. In addition, R263 directly regulates the vDNA catalytic loop geometry by forming a dual hydrogen bond to the N144 residue positioned at the N terminus of this loop. Thus, the HIV-1 intasome structure containing the wild-type R263 amino acid revealed coordinated “cross talk” between the catalytic site via the vDNA catalytic loop and the 3′ and 5′ termini of the vDNA, which was more complicated than previously hypothesized. However, with the R263K substitution, multiple hydrogen bonds with the substrate and/or vDNA catalytic loop are eliminated, providing additional flexibility and disrupting the cross talk between the HIV-1 integrase and both vDNA termini. The additional flexibility in the catalytic loop affects the binding kinetics of vDNA, tDNA, and, as we have determined experimentally, INSTIs, with substrates having a higher binding preference over INSTIs. Modulation of the coordinated cross talk between the vDNA and the catalytic site via the vDNA catalytic loop likely decreases viral replication capacity as a result of geometric changes in the catalytic site relative to the wild type.

The structural and electronic characteristics of dolutegravir likely confer the prolonged binding to wild-type and mutant integrase-DNA complexes and therefore underpin its high barrier to resistance (4, 35). The HIV-1 fitness landscape varies based on numerous drug characteristics, including barrier to resistance (39, 40). For drugs with a lower barrier, effective resistance can be achieved with a single mutation while maintaining viral fitness; for example, efavirenz resistance can occur via the single reverse transcriptase substitution K103N (41). In contrast, for drugs with a higher resistance barrier, a single mutation may only confer low levels of effective resistance and/or cause decreased viral fitness, which may lead to an evolutionary dead end. The accumulation of G118R and R263K and additional substitutions in this study can reflect a difficult pathway toward achieving dolutegravir resistance that is also associated with a loss in viral fitness. Traversal of this HIV fitness landscape as in the SAILING, DAWNING, and P1093 studies (1, 9, 32) is likely facilitated by limited background regimen options and via difficulties with adherence, which decrease the pharmacokinetic component of a resistance barrier.

Nonadherence and weak regimen support are common reasons for treatment failure (42). While some viral load progressions in this study showed transient elevations consistent with nonadherence, no direct measurements of drug levels were gathered in the DAWNING study. Acquisition of integrase substitutions in DAWNING was associated with increased dolutegravir resistance and reduced replication capacity in all participants. Most participants had integrase substitutions of G118R, either alone or in combination with other substitutions, including R263R/K. G118R alone had a greater impact on increasing dolutegravir resistance and reducing replication capacity compared with G118R plus other integrase substitutions. Dolutegravir retained prolonged binding to integrase-DNA complexes with G118R or R263K substitutions relative to other INSTIs, and the drug’s resistance through this pathway could be rationalized at the HIV-1 integrase molecular level. Overall, the results here provide underpinning rationale for the high barrier to resistance of dolutegravir and indicate that *de novo* resistance is not straightforward and apparently comes at the expense of reduced viral fitness.

## MATERIALS AND METHODS

### Study design.

DAWNING is a multicenter, open-label, parallel-group, randomized, active-controlled, noninferiority phase IIIb study evaluating dolutegravir compared with ritonavir-boosted lopinavir as second-line ART in adults with HIV-1 infection (ClinicalTrials.gov identifier NCT02227238) (9). Eligible study participants were ≥18 years old, had been treated with a first-line regimen consisting of 1 NNRTI plus 2 NRTIs for ≥6 months, and were experiencing virologic failure (HIV-1 RNA levels of ≥400 copies/mL on 2 consecutive visits ≥7 days apart) at screening. Participants were randomized 1:1 to receive 50 mg dolutegravir once daily or ritonavir-boosted lopinavir at doses of either 800 mg lopinavir with 200 mg ritonavir once daily or 400 mg lopinavir with 100 mg ritonavir twice daily, each administered in combination with an investigator-selected background regimen consisting of 2 NRTIs, including ≥1 fully active NRTI.

Confirmed virologic withdrawal criteria were defined as 2 consecutive measurements with a decrease in plasma HIV-1 RNA of <1.0 log_10_ copies/mL by week 16 (unless HIV-1 RNA levels were <400 copies/mL), plasma HIV-1 RNA levels of ≥400 copies/mL at or after week 24, or plasma HIV-1 RNA levels of ≥400 copies/mL after confirmed HIV-1 RNA levels of <400 copies/mL. Participants who met CVW criteria were withdrawn from the study. For this *ad hoc* assessment carried out after the week 48 analysis, participants with available data through 12 September 2019 were included. The primary week 48 analysis had a cutoff date of 2 August 2017 for week 52 last-participant last-visit data. The data from this *post hoc* analysis were based on a not fully cleaned or locked database.

The study was conducted in accordance with local regulatory requirements and the International Conference on Harmonization of Technical Requirements for Registration of Pharmaceuticals for Human Use Good Clinical Practice following the principles of the 2008 Declaration of Helsinki. The study protocol was reviewed and approved by national, regional, or investigational center ethics committees or institutional review boards. All participants provided written informed consent and could voluntarily withdraw from the study at any time.

### Population resistance testing.

HIV-1 genotyping and phenotyping of plasma samples at baseline and the first sample of the 2 required to meet CVW criteria were conducted by Monogram Biosciences (South San Francisco, CA) using PhenoSense Integrase, PhenoSense GT, and GeneSeq Integrase assays, with the PhenoSense GT Plus Integrase assay used as a backup alternative in case of initial resistance assay failure. Integrase region-based replication capacity results are from the PhenoSense Integrase assay and indicate the ability of recombinant viruses containing participant-derived integrase sequences to replicate in the absence of drug compared with wild-type HIV-1.

### Clonal genotyping and phylogenetic analyses.

Clonal analyses used the same samples and time points as were used for the corresponding population resistance testing. Integrase genotyping and phenotyping for dolutegravir, elvitegravir, and raltegravir were assessed on ≥8 variant clones at each time point by Monogram Biosciences for participants who originally met CVW criteria in the week 48 analysis. Phylogenetic analysis of 64 clonal and 6 population integrase nucleotide sequences was performed. A maximum-likelihood tree was created using the IQ-TREE application with TN+F+G4 plus Gamma modeling, and a K clade sample was used as the outgroup (43). Evolutionary distance branch support was determined using 1,000 bootstrap replicates.

### INSTI dissociation analyses.

Dissociation of INSTIs from integrase-DNA complexes was evaluated as previously described (35). Briefly, biotinylated viral long-terminal-repeat DNA duplexes were prepared and attached to streptavidin-coated scintillation proximity assay imaging beads. Wild-type and R263K, G118R, or G118R/E138K mutant integrase-DNA-bead complexes were formed, and unbound protein was removed. Integrase-DNA-bead complexes or control DNA-bead complexes were mixed with ^3^H-labeled dolutegravir, elvitegravir, or raltegravir in a 96-well microplate, and dissociation of ^3^H-labeled INSTIs at 37°C was monitored for up to 3 weeks using a ViewLux charge-coupled device imager (PerkinElmer, Waltham, MA). Relative binding (RB) was calculated as the ratio of dissociation signal to high signal and fit with the equation RB = EP + ΔRB(*e*^−^*^k^*^off^*^t^*), where EP is the RB endpoint, *k*_off_ is the dissociation rate constant, ΔRB is the change in RB, and *t* is time, using the SigmaPlot program (Systat Software, San Jose, CA). Natural logarithm of 2 divided by mean *k*_off_ value was used to calculate dissociative half-life.

### HIV-1 integrase structural analyses.

The cryo-EM structure of the HIV-1 intasome was downloaded from the Research Collaboratory for Structural Bioinformatics (Protein Data Bank [PDB] ID 5U1C) in PDB file format (11). The protein-substrate complex was prepared using Maestro software (version 12.1.013; Schrödinger LLC, New York, NY) by adding hydrogens to all heavy atoms present in the complex (44, 45). The G118 residues in both HIV-1 integrase chains A and C were selected for mutations because both integrase chains are shown to be directly involved in the integration of either vDNA or tDNA substrates in the cryo-EM structure. The “Mutate Residue” function was used to convert the glycine (G) to arginine (R) for the selected G118 residues. The “Select Rotamer” function was then used to select a conformation of the G118R mutant residue that best interacted with the 3′ hydroxy of the tDNA terminal thymidine (base 11) in chain G, while forming a hydrogen bonding network with E92 in chain A of the HIV-1 integrase catalytic core. A similar procedure was used for the G118R mutant residue in chain C. For G118R residues in chains A and C, a localized Prime calculation was executed to optimize the interaction of G118R with both E92 and the 3′ hydroxy of the tDNA terminal thymidine (46–48). The resulting coordinates were captured in Maestro software, and the hydrogens were removed from both proteins and substrates. The resulting complex was exported as a PDB file for further analysis, and images were created with the PyMOL molecular graphics system (version 1.7.6.6; Schrödinger LLC).

The protein/substrate complex with mutations of the R263 residue was constructed from the HIV-1 intasome structure (PDB ID 5U1C) in a manner similar to that for the G118R mutant protein (11). The “Mutate Residue” function in the Maestro software was used to convert the arginine (R) to lysine (K) for the selected R263 residues in HIV-1 integrase chains A and C. The “Select Rotamer” function was then used to select a conformation of the R263K mutant residue that best interacted with the 5′ phosphate of the vDNA adenosine (base 18) in chain F while maintaining a conformation similar to the wild-type R263 amino acid side chain. A similar procedure was used for the R263K mutant residue of chain C. The resulting R263K homology model was then used as a starting geometry for the construction of a second R263K model containing an alternate conformation of this residue. The “Select Rotamer” function was used to select a conformation of the R263K mutant residue in chain A from the model in which R263K best interacted with both N144 and Q146 in chain A while maintaining a conformation similar to the wild-type R263 amino acid side chain. A similar procedure was used for the R263K mutant residue in chain C. The resulting coordinates for both R263K homology models were captured in Maestro software, and the hydrogens were removed from both proteins and substrates. The resulting complex was exported as a PDB file for further analysis, and images were created using PyMOL software.

### Data availability.

Clonal and population sequences have been deposited in GenBank under accession numbers MZ568467 through MZ568625. Anonymized individual participant data and study documents can be requested for further research from www.clinicalstudydatarequest.com.
