# Supplementary material for: Integrase Inhibitor Resistance Mechanisms and Structural Characteristics in Antiretroviral Therapy-Experienced, Integrase Inhibitor-Naive Adults with HIV-1 Infection Treated with Dolutegravir plus Two Nucleoside Reverse Transcriptase Inhibitors in the DAWNING Study
Source: Antimicrob Agents Chemother. 2022 Jan 18;66(1):e01643-21. doi: 10.1128/AAC.01643-21 (PMC8765460; doi:10.1128/AAC.01643-21)
Supplement: Supplemental file 1 — Supplemental Text S1. Download AAC.01643-21-s0001.pdf, PDF file, 0.1 MB [file aac.01643-21-s0001.pdf]

Supplemental File S1

Integrase Inhibitor Resistance Mechanisms and Structural Characteristics in Antiretroviral Therapy–Experienced, Integrase Inhibitor–Naive Adults With HIV-1 Infection Treated With Dolutegravir Plus 2 Nucleoside Reverse Transcriptase Inhibitors in the DAWNING Study

Mark Underwood,[a] Joe Horton,[b] Keith Nangle,[b] Judy Hopking,[c] Kimberly Smith,[a] Michael Aboud,[d] Brian Wynne,[a] Jörg Sievers,[d] Eugene L. Stewart,[e] Ruolan Wang[a]

[a]ViiV Healthcare, Research Triangle Park, NC, USA; [b]Parexel International, Durham, NC, USA; [c]GlaxoSmithKline, Stockley Park, UK; [d]ViiV Healthcare, Brentford, UK; [e]GlaxoSmithKline, Upper Providence, PA, USA

Background

HIV-1 integrase structural analyses described fully in the manuscript were carried out by either examination of the cryogenic electron microscopy (cryo-EM) structure of the HIV-1 intasome[1] or—in the case of dolutegravir (DTG)-bound HIV-1 INT—the cryo-EM structure of SIV intasome bound with DTG[2] downloaded from the Research Collaboratory for Structural Bioinformatics (protein data bank [PDB] ID: 5U1C and 6RWN, respectively) in PDB file format. Any mutant HIV-1 INT structures illustrated here were computationally derived from the cryo-EM HIV-1 intasome structure as described in the manuscript.

Scene 1 (0:04)

Cartoon and stick rendering of HIV-1 strand transfer complex intasome structure (also see Passos et al 2017,[1] Figure 1B).

The HIV-1 INT proteins are displayed in cartoon and colored magenta, while both the viral DNA (vDNA) and target DNA (tDNA) are in stick and colored orange. The intasome complex is composed of two pairs of dimeric HIV-1 INT proteins encompassing the 3’- and 5’-strands of both vDNA termini (shown vertically through the middle of the complex) and one double strand of the tDNA (horizontal near the bottom of the complex). The rendered green sphere is the catalytic Mg^{2+} and added for the purpose of identifying the catalytic pocket.

→ *Rotation and zoom in occurs to Scene 2*

Scene 2 (0:14)

View is perpendicular to the vDNA helical axis through to the catalytic Mg^{2+} and illustrating the catalytic/DTG-binding pocket. This perspective is orthogonal to the view of DTG and the HIV-1 INT catalytic pocket as shown in Figure 4A of the manuscript.

The surface of the HIV-1 INT catalytic site is rendered in transparent gray, with DTG oriented in that pocket, rendered in ball and stick, and colored by atom with carbons in white, oxygens in red, and nitrogens in blue. The nucleic acid components of the catalytic site are labeled as viral 5’-guanosine, viral 3’-cytidine, and viral 3’-adenosine, as are specific protein chain amino acids Y143, N144, and P145. Two Mg^{2+}s are rendered as green spheres and illustrate the binding of DTG to these Mg^{2+}s via its dual carbonyl oxygens.

→ *Structure rotates to Scene 3*

Scene 3 (0:19)

This view shows the vDNA and tDNA 3'-terminal nucleotides bound to **wild-type** G118. The cartoon and stick rendered protein is in magenta, and vDNA and tDNA substrates are shown in orange backbone with the terminal nucleotides rendered in stick and colored magenta. The green Mg^{2+} sphere and hydrogen bond interactions (yellow dashed lines) to the catalytic amino acid residues—D64 and D116—are shown with those residues depicted as sticks.

→ *Structural changes are observed when wild-type G118 is replaced with R118 in Scene 4*

Scene 4 (0:24)

The single-mutant R118 HIV-1 INT is colored orange and rendered in cartoon with key residues in the protein and substrates rendered as sticks. The color of the protein has been modified to highlight gross structural changes that occur when the wild-type G118 is replaced with R118 (colored by atom in cyan). Hydrogen bonds between the Mg^{2+} and catalytic amino acids—D64 and D116—remain (yellow dashed lines); however, an additional dual hydrogen bonding interaction with E92 (colored by atom in cyan) is observed and stabilizes the R118 substitution, facilitating an additional hydrogen bond with the 3'-hydroxy group of tDNA terminal thymidine.

→ *Structure rotates transiently to original Scene 1 and then to Scene 5*

Scene 5 (0:33)

This view illustrates the proximity of R263 to the HIV-1 INT catalytic binding site identifiable by the catalytic Mg^{2+} (rendered as a green sphere) and the catalytic amino acids—D64 (unlabeled) and D116—both rendered as sticks and colored in magenta (as is the cartoon rendering of the protein). The HIV-1 INT vDNA catalytic loop is colored in cyan to highlight its relative position to R263. R263 (rendered as sticks and colored cyan) is hydrogen bonded to both the 3'- and 5'-strands of the vDNA and amino acid N144. This perspective clearly illustrates the role of R263 in regulating the position of the catalytic loop in the presence of the 3'- and 5'-strands of the vDNA, thus stabilizing the arrangement of viral substrate for strand transfer at both termini. The catalytic Mg^{2+} can be observed hydrogen bonded to the catalytic residue D116 now positioned near the bottom of the scene and in proximity to the terminal viral 3'-adenosine.

Scene 6 (0:37)

DTG has been added transiently to Scene 5 for comparison with the unbound HIV-1 INT. Note that while DTG is relatively distal from R263, the binding pocket occupied by the inhibitor is in part formed by the vDNA catalytic loop (which contains N144) and the viral 5'-guanosine. As both N144 and the viral 5'-guanosine are directly interacting with R263, this residue thereby has indirect influence over the geometry of the inhibitor binding pocket and ultimately the drug's ability to bind to HIV-1 INT.

→ *Next R263 wild-type is replaced with mutant K263, with protein backbone changed from magenta to green to visualize structural changes from Scene 6 to Scene 7*

Scene 7 (0:42)

K263 (colored by atom in deep magenta) now only hydrogen bonds with the 3'-strand of the vDNA with the loss of the complex hydrogen bonding system between the catalytic loop (colored in deep magenta) and the viral 5'-guanosine. The resulting decoupling of the cross-talk

between the two termini of the vDNA results in geometrical changes in both the position of the catalytic loop and the viral 5'-guanosine and ultimately the geometry of the inhibitor binding pocket. These changes are predicted to result in the inability of DTG to bind effectively to that pocket and optimally inhibit strand transfer.

Scene 8 (0:45)

DTG has been added transiently to Scene 7 for comparison with the unbound K263 mutant HIV-1 INT. The Mg^{2+} can still be seen in the lower part of the scene rendered as a green sphere.

→ DTG is then removed and the structure rotates to Scene 9 to display integrase amino acid positions 118 on the left, the Mg^{2+} in proximity to 118, and the distal position of amino acid position 263 (shown on the right side of the scene) relative to 118

Scene 9 (0:52)

See also Manuscript Figure 4B and 4C and supporting text for additional descriptions and details.

Initially, the R118 mutation (colored by atom in pink) is shown with R263 wild-type (colored by atom in orange) and multiple stabilizing hydrogen bonds with R263. The tDNA and vDNA are presented in dark orange. The HIV-1 INT tDNA catalytic loop is colored in pink, and the vDNA catalytic loop is colored in magenta. Note the close proximity of the two catalytic loops and how the interaction of R118 (on the tDNA catalytic loop) and R263 (via its hydrogen bonding with N144 on the vDNA catalytic loop) provide an effective yet distal cross-talk between the two residues leading to dual modulation of the protein's catalytic function.

→ tDNA 3'- and 5'-strands are removed and DTG is added for Scene 10

Scene 10 (0:56)

DTG is positioned and added transiently to illustrate its binding between the vDNA and tDNA catalytic loops and interaction with the catalytic Mg^{2+}. Hydrogen bonding interactions between N144 with R263 and R118 with E92 are indicated by dashed yellow lines.

DTG is removed and the vDNA- and tDNA-bound HIV-1 INT is shown during transition to the next scene. The mutant R118 is replaced with wild-type G118 (colored by atom in pink), and R263 is replaced with mutant K263 (colored by atom in deep magenta) to illustrate the relative position of these two residues and their influence on the two catalytic loops.

Scene 11 (1:00)

This view is identical to that of Scene 9; however, the wild-type G118 and mutant K263 are illustrated. The HIV-1 INT tDNA and vDNA catalytic loops are highlighted in pink and magenta, respectively, and illustrate the locations of G118 and N144 (rendered in stick) on each loop.

→ Next the tDNA 3'- and 5'-strands are removed and DTG is transiently added for Scene 12

Scene 12 (1:04)

This view is identical to that of Scene 10 (with DTG transiently positioned in the putative binding site); however, the wild-type G118 and mutant K263 are illustrated here. The integrase G118

catalytic site and N144 are shown with the K263 mutant illustrating a more limited overall hydrogen bonding network—as indicated by dashed yellow lines—throughout the protein.

→ *Structure rotates back to original Scene 1*

References

1. Passos DO, Li M, Yang R, Rebensburg SV, Ghirlando R, Jeon Y, Shkriabai N, Kvaratskhelia M, Craigie R, Lyumkis D. 2017. Cryo-EM structures and atomic model of the HIV-1 strand transfer complex intasome. Science 355:89–92.
2. Cook NJ, Li W, Berta D, Badaoui M, Ballandras-Colas A, Nans A, Kotecha A, Rosta E, Engelman AN, Cherepanov P. 2020. Structural basis of second-generation HIV integrase inhibitor action and viral resistance. Science 367:806–810.
